# Multiomics in Renal Cell Carcinoma: Current Landscape and Future Directions for Precision Medicine

**DOI:** 10.1007/s11934-025-01276-2

**Published:** 2025-05-26

**Authors:** Filippo Gavi, Maria Chiara Sighinolfi, Giuseppe Pallotta, Simone Assumma, Enrico Panio, Daniele Fettucciari, Antonio Silvestri, Pierluigi Russo, Riccardo Bientinesi, Nazario Foschi, Filippo Turri, Umberto Carbonara, Chiara Ciccarese, Roberto Iacovelli, Camilla Nero, Bernardo Rocco

**Affiliations:** 1 Department of Urology, Fondazione Policlinico Universitario Agostino Gemelli, IRCCS, Rome, 00168 Italy; 2 Unit of Urology, Policlinico di Bari, Bari, Italy; 3 Department of Oncology, Fondazione Policlinico Universitario Agostino Gemelli, IRCCS, Rome, 00168 Italy; 4 Department of Women and Child Health, Fondazione Policlinico Universitario Agostino Gemelli, IRCCS, Rome, 00168 Italy

**Keywords:** Kidney cancer, Renal cell carcinoma, Clear cell carcinoma, Omics sciences

## Abstract

**Purpose of Review:**

Renal cell carcinoma (RCC) is a prevalent and increasingly diagnosed malignancy associated with high mortality and recurrence rates. Traditional diagnostic and therapeutic approaches have limitations due to the disease’s molecular heterogeneity. This review aims to explore how the integration of omics sciences—genomics, transcriptomics, proteomics, and metabolomics—can enhance the diagnosis, prognosis, and treatment of RCC.

**Recent Findings:**

Genomic analyses have uncovered critical mutations, including VHL, PBRM1, and BAP1, which support improved risk stratification and the development of targeted therapies. Transcriptomic and spatial transcriptomic studies have provided deeper insights into RCC heterogeneity and tumor microenvironment dynamics. Proteomic investigations have revealed potential biomarkers, while metabolomic approaches have highlighted RCC-specific metabolic shifts. Despite these advancements, several challenges persist, including intratumoral heterogeneity, difficulties in multi-omics data integration, and the limited clinical validation of biomarkers.

**Summary:**

Omics-driven approaches hold significant promise for advancing precision medicine in RCC. These technologies can facilitate earlier diagnosis, guide individualized therapies, and enhance prognostic evaluations. Future research must focus on validating multi-omic biomarkers and leveraging artificial intelligence to manage complex datasets, thereby supporting more informed clinical decision-making and personalized treatment strategies.

## Introduction

Renal cell carcinoma (RCC) is one of the most prevalent malignancies worldwide, with approximately 300,000 new diagnoses and 100,000 deaths each year [1]. The highest incidence rates are observed in North America and Europe, with a 2% increase in RCC incidence per year over the past twenty years [2]. RCC occurs more frequently in men and can be either hereditary or sporadic, with genetic mutations commonly playing a key-role [3]. Mortality remained unchanged since 1990 even though it slightly decreased [4]. Interestingly, a number of modifiable risk factors have been found, including cigarette smoking, obesity, and hypertension [5]. Nonetheless, no validated screening programs are in place due to a lack of studies reporting its efficacy, cost-effectiveness, and optimal modality [6]. Besides these histological types, there are 10% of very uncommon, sporadic, familiar, and non-classified RCC [7]. RCC remains a significant health burden, characterized by high mortality and recurrence rates.

In the metastatic setting, immunotherapy or a combination of immunotherapy and a tyrosine kinase inhibitor are the best approach. Even with systemic therapy, 75% of patients are resistant and those who initially respond can progress. However, currently there are not effective biomarkers or molecular classification system to tailored treatments and follow up for each RCC patient. RCC is known for its heterogeneity due to its molecular differences [8]. The developing of multi omics analysis from RCC patients, have permit a better understanding of the molecular pathways and characteristics [9]. Combining multi omics analysis and artificial intelligence algorithm can offer newer prognostics indicators predicting treatment response [10]. Improved diagnostic techniques and a deeper understanding of RCC biology are essential for reducing overall mortality and advancing patient-specific treatments. Herein, we present a comprehensive review of the literature aiming to summarize the current evidence regarding the integration of “omics” sciences, intended as the combination of data from different ‘omics’ layers to achieve a comprehensive understanding of biological systems, to enhance the management of RCC.

## Methods

A comprehensive literature review was conducted using PubMed, Scopus, and Web of Science databases. The search strategy included studies published between 2000 and 2025, focusing on the role of omics sciences in RCC. Inclusion criteria encompassed studies investigating genetic and molecular alterations, novel biomarkers, and the application of multi-omics approaches in RCC management. Articles were assessed for quality and relevance based on study design, sample size, and clinical applicability.

## Renal Cell Carcinoma Diagnosis and Treatment

Patients with RCC only present symptoms in advanced stages [11]. The majority of RCC are detected incidentally with ultrasound (US) or because of nonspecific symptoms correlated to other abdominal conditions [12]. US, computed tomography (CT) and magnetic resonance imaging (MRI) is the imaging technique for mass characterization [13].

In terms of treatment, surgery is the only curative treatment for localized RCC Localized T1. RCCs are best treated with partial nephrectomy (PN) rather than radical nephrectomy (RN) [14]. PN is associated with better preservation of general kidney function and should be offered whenever feasible [15]. When surgery is not possible other options include targeted therapy immunotherapy, radiotherapy, ablation, or a combination of these treatments [16]. Metastatic kidney cancer patients frequently receive several types of treatment including surgery, radio, and immunotherapy [17]. These techniques are time-consuming, labor-intensive, and not cost-effective [18]. In recent years studies on RCC biology have disclosed new methods and new molecular approaches for diagnosis and treatment. The use of omics revolutionized the way of thinking about RCC. Genomics and sequencing have linked VHL (64%), SETD2 (20%), and BAP1 (13%) as the most frequent genes involved in RCC [19–21]. Transcriptomics provided novel insights for biomarkers and identifying renal cell cancer subtypes improving detection and tailored therapy [22–24]. Proteomics has exposed novel soluble protein biomarkers and exosomes that are promising for RCC treatment and are available from blood and urine [25, 26]. Biomarkers for RCC have also been demonstrated by proteomics analysis and could be used in the future [27]. Metabolomics has shown new biochemical pathways that could explain RCC adaptation not only aberration in the cancer itself but also normal pathways used for the cancer’s needs [28]. Most frequent omics sciences are reported in Table 1.

**Table 1:** Most frequent omics science

Omics sciences	Omics type	Method	Concept
Genomics	DNA or RNA	Genome-Wide Identification of SNPs Linked to Clinical Traits	Genotyping Arrays and Whole Exome Sequencing for Variant Detection
Metabolomics	Metabolites	Metabolites expression	Mass Spectrometry-Based Techniques for Molecular Profiling
Proteomics	Protein	Protein expression	Mass Spectrometry and Antibody-Based Strategies for Biomolecular Analysis
Transcriptomics	RNA	Expression of mRNA	Microarray Analysis and RNA Sequencing

Notes: SNPs: Single Nucleotide Polymorphisms

## Applications of Omics Sciences in Renal Cell Carcinoma

Kidney cancer is a complex disease characterized by significant variability and heterogeneity, as demonstrated through various analytical methods [29]. Moreover, the integration of omics sciences allowed the highlighting of novel patterns and molecular data on RCC biology, including pathologic alteration, cellular growth, and response to treatments [30, 31]. Researchers can now investigate molecular and genetic variations using advanced techniques like spatial transcriptomics and spatial proteomics. These methods enable them to map genetic and cellular differences across both healthy and cancerous tissues while preserving the tissue’s structural integrity [32]. Omics sciences also facilitate the discovery of new drug targets and biomarkers, aiding in early diagnosis and enabling healthcare professionals to generate comprehensive metabolic profiles for each patient, who can then receive biologically tailored treatments. However, several challenges arise when applying omics technologies in diagnostics and therapy, particularly due to tumor heterogeneity, which complicates the identification of biomarkers that truly represent the entire tumor [24]. Additionally, the acquisition and analysis of adequate tissue samples require the integration of data from multiple omics platforms. Most importantly, there remains a shortage of prognostic molecular biomarkers for advanced kidney cancer [29, 33, 34].

## Genomics and Genetics in Renal Cell Carcinoma

Genetic mutations are associated with an increased risk of presenting RCC. Several mutations have been found through genomic sequencing [35, 36]. The risk of developing RCC varies depending on the type of genetic predisposition. VHL, PBRM1, BAP1, SETD2, and KDM5C are the most frequent mutated genes in RCC [37]. Genes related to RCC are reported in Table 2. Although multiple mutations can occur in RCC, most of them are associated with a wide range of genetic variations. Most of these variations involve coding mutations, including missense, nonsense, and insertion/deletion changes, which appear at different frequencies across major genetic syndromes [38]. Research on these mutations has provided new insights into treatment response mechanisms in RCC, contributing to the development of more personalized therapies. While biomarkers are being actively investigated, none have yet received approval for clinical use in RCC [39].

**Table 2:** Gene correlated with RCC

Gene	Gene loci	Histology	Inheritance
VHL	*3p25.3*	RCC	AD
PTEN	*10q23.31*	ccRCC	AD
MET	*7q31.2*	RCC, papillary, familial and somatic	AD
FH	*1q43*	Leiomyomatosis and RCC	AD
FLCN	*17p11.2*	RCC, chromophobe, comatic	AD
BAP1	*3p21.1*	RCC	Driver gene
CHEK2	*22q12.1*	RCC	-
CDC73	*1q31.2*	ccRCC	-
PBRM1	*3p21.1*	RCC, ccRCC	-
OGG1	*3p25.3*	RCC, ccRCC	-
RNF139	*8q24.13*	RCC	-
HNF1A	*12q24.31*	RCC	-
HNF1B	*17q12*	RCC	-

Notes: AD: autosomic dominant; ccRCC: clear cell carcinoma; RCC: renal cell carcinoma

Genomics has improved our understanding of response mechanisms, particularly in targeted therapies or oral multi-targeted tyrosine kinase inhibitors (TKIs) for metastatic RCC [40]. Sorafenib and sunitinib were the first, followed by axitinib, tivozanib, and cabozantinib [41]. In recent years, genomics has been increasingly applied to the study of circulating tumor DNA (ctDNA) in plasma. Liquid biopsy offers a valuable tool for cancer diagnosis and predicting recurrence. Moreover, the genomic profiling of ctDNA has been shown to reflect tumor heterogeneity, playing a pivotal role in identifying resistant clones and informing the selection of targeted therapies well before clinical or radiographic changes become evident, particularly in bladder cancer, but still in RCC is under research [42].

## Metabolomics in Renal Cell Carcinoma

Pathway analysis of RCC tissues has revealed the activation of angiogenesis, glycolysis, and immune-related pathways, while simultaneously highlighting the suppression of various metabolic pathways. These findings provide valuable insights into the molecular mechanisms driving RCC progression and may contribute to the identification of novel therapeutic targets and biomarkers for improved patient management [10].

## Proteomics in Renal Cell Carcinoma

The advent of large-scale sequencing technologies has significantly advanced the genetic profiling of RCC. This has led to the discovery of mutations in several genes not previously linked to clear cell tumors. Among these, PBRM1—encoding BAF180, a key chromatin-targeting subunit of the SWI/SNF chromatin remodeling complex—emerged as one of the most frequently altered genes after VHL [43]. The mRNA gene expression analysis has been widely utilized to identify differences between normal and malignant renal tissues. This approach has also been instrumental in developing gene signatures associated with various RCC subtypes, as well as prognostic and metastatic potential [44, 45]. Although proteomics studies in RCC have yielded promising results, further research with larger sample sizes and more comprehensive proteomic coverage is necessary to facilitate the translation of these findings into clinical practice [46]. A list of proteomics biomarkers is summarized in Table 3.

**Table 3:** Proteomics biomarkers correlated to RCC

Biomarker	Protein class	Expressed
CAIX	Enzymes, metabolic proteins	ccRCC
KIM-1	CD markers	RCC
PLIN2	Metabolic proteins, Plasma proteins	RCC
RKIP	Plasma proteins	Marker in RCC
VEGF	PDGF/VEGF growth factor family	RCC

Notes: ccRCC: clear cell carcinoma; RCC: renal cell carcinoma

## Transcriptomics and Spatial Transcriptomics in Renal Cell Carcinoma

Although nearly all human cells share an almost identical genome, they develop into distinct cell types through variations in gene expression. These differences arise from complex epigenetic modifications, which regulate which genes are activated or silenced. Such modifications, including DNA methylation and histone modifications, guide cellular differentiation, allowing specialized functions to emerge despite genetic uniformity [47]. The application of single-cell and spatial transcriptomics in renal cell carcinoma (RCC) has significantly advanced our understanding of its cellular and molecular heterogeneity. These cutting-edge technologies have enabled unprecedented insights into the tumor microenvironment (TME) of clear cell RCC (ccRCC), elucidating the complex spatial organization and interactions between malignant and non-malignant cells. Li et al. (2022) [48] mapped over 270,000 single-cell transcriptomes and demonstrated that the intratumoral heterogeneity of CD8⁺ T cell clonotypes exceeds that of somatic mutations, while also revealing an enrichment of epithelial-mesenchymal transition (EMT) at the tumor-normal interface co-localized with IL1B-expressing macrophages—potential targets for therapy [48]. Complementing these findings, Yang et al. (2024) highlighted metabolic heterogeneity within ccRCC by correlating CD8⁺ T cell energy metabolism with exhaustion and linking sphingolipid metabolism to M2 macrophage polarization; notably, they identified that the tumor epicenter exhibits heightened metabolic activity, with increased tricarboxylic acid cycle engagement and glycolysis, whereas the pericapsular regions are enriched in markers of vasculogenesis, inflammatory responses, and EMT [49]. Moreover, Song et al. (2025) uncovered a novel subset of cancer-promoting regulatory T (Treg) cells expressing IL-1β and IL-18, which interact with MRC1⁺FOLR2⁺ tumor-associated macrophages at the tumor-normal interface, thereby reinforcing an immunosuppressive TME that fosters tumor progression [50]. Together, these integrated approaches not only deepen our understanding of ccRCC spatial and functional heterogeneity but also pave the way for the identification of novel therapeutic targets.

## Multiomics, Panomics and Future Prospectives

Recent advances in multi-omics technologies have significantly enhanced our understanding of renal cell RCC, particularly ccRCC, through the integration of genomic, transcriptomic, epigenomic, proteomic, and metabolomic data. Initial multi-omics analyses highlighted the crucial role of VHL gene inactivation in driving angiogenic and proliferative pathways [51, 52]. Subsequent pan-omics studies have further refined our knowledge of RCC subtypes and metastatic mechanisms. For instance, Hu et al. [10] identified distinct immune-metabolic subtypes in ccRCC, characterized by varying immune infiltrates and metabolic signatures, which correlate with patient prognosis. Additionally, Chen et al. [53] pinpointed SERPINE2 as a key driver of metastasis through multi-omics integration, revealing its involvement in epithelial-mesenchymal transition (EMT). These multi-omics findings underscore the potential for improved tumor subclassification, identification of therapeutic targets, and the development of precision medicine strategies in RCC. Looking ahead, the landscape of RCC research stands poised to enter a new era, where the integration of advanced omics technologies will revolutionize clinical practice. Building upon the foundational strides made in transcriptomics, metabolomics, and immunogenomics, the future will witness the seamless convergence of multi-omics and radiogenomics to create comprehensive patient profiles. This pan-omics approach promises to transcend current limitations, enabling the precise identification of predictive biomarkers that guide personalized, mechanism-driven combination therapies. The anticipated era heralds a deeper understanding of RCC heterogeneity and resistance mechanisms and the potential to preemptively tailor treatments, ultimately transforming RCC management from a reactive to a proactive strategy [54]. The ambition is clear: to leverage the power of integrated omics to deliver a future where individualized, data-driven interventions become the standard of care, significantly improving patient outcomes across all RCC subtypes.

## Conclusions

Renal cell carcinoma remains a significant global health challenge, characterized by high incidence, mortality, and heterogeneity. Advances in omics sciences including genomics, transcriptomics, proteomics, and metabolomics have provided unprecedented insights into RCC biology, paving the way for precision medicine approaches. The integration of these technologies has enhanced our ability to identify novel biomarkers, understand tumor progression, and develop targeted therapies. Despite these advancements, several challenges persist, particularly in translating omics findings into routine clinical practice. Tumor heterogeneity, the complexity of data integration, and the need for large-scale validation studies remain key hurdles. However, the future of RCC management is increasingly moving towards a multi-omics, personalized approach, where predictive and prognostic biomarkers will guide tailored therapies, improving patient outcomes. As research continues to evolve, the synergy between omics technologies and emerging fields such as radiogenomics and artificial intelligence holds the potential to further refine RCC diagnosis, risk stratification, and treatment selection. Ultimately, the goal is to shift from a reactive to a proactive model of care, ensuring that each patient receives the most effective, individualized treatment strategy.

## Data Availability

No datasets were generated or analysed during the current study.

## Key References

- Eismann L, Xie AX, Tang C, Knezevic A, Ostrovnaya I, Kuo F, et al. *Sample Site Impacts RNA Biomarkers for Renal Cell Carcinoma.* Eur Urol. 2025 Jan;87(1):79–83.
  - • Important: Highlights the critical influence of tissue sampling location on RNA biomarker reliability, underscoring a key methodological consideration for transcriptomic studies in RCC.
- Bui TO, Dao VT, Nguyen VT, Feugeas JP, Pamoukdjian F, Bousquet G. *Genomics of Clear-cell Renal Cell Carcinoma: A Systematic Review and Meta-analysis.* Eur Urol. 2022 Apr;81(4):349–61.
  - •• Very Important: Provides a comprehensive synthesis of genomic alterations in clear-cell RCC and supports stratification frameworks; serves as a foundational resource for precision oncology in RCC.
    • Additionally, the meta-analysis enhances understanding of mutation prevalence and potential therapeutic targets across diverse populations.
- Genomic and Transcriptomic Predictors of Response from Stereotactic Body Radiation Therapy in Patients with Oligoprogressive Renal Cell Carcinoma. Eur Urol Oncol. 2023 Aug;6(4):447–50.
  - • Important: Demonstrates the role of integrated omics in predicting therapeutic response, supporting the clinical utility of genomics and transcriptomics in treatment selection for advanced RCC.
- Figiel S, Bates A, Braun DA, Eapen R, Eckstein M, Manley BJ, et al. *Clinical Implications of Basic Research: Exploring the Transformative Potential of Spatial ’Omics in Uro-oncology.* Eur Urol. 2025 Jan;87(1):8–14.
  - •• Very Important: Emphasizes the emerging impact of spatial omics in characterizing tumor heterogeneity and microenvironmental interactions, which is pivotal for developing context-specific RCC therapies.
    • Bridges translational research with clinical practice, advocating for the adoption of spatial omics in personalized uro-oncologic care.
